# Diabetes treatment patterns and goal achievement in primary diabetes care (DiaRegis) - study protocol and patient characteristics at baseline

**DOI:** 10.1186/1475-2840-9-53

**Published:** 2010-09-16

**Authors:** Peter Bramlage, Christiane Binz, Anselm K Gitt, Michael Krekler, Tanja Plate, Evelin Deeg, Diethelm Tschöpe

**Affiliations:** 1 Institute for Cardiovascular Pharmacology and Epidemiology, Mahlow, Germany; 2 Bristol Myers Squibb, Medical Department, Munich, Germany; 3 Institut für Herzinfarktforschung Ludwigshafen an der Universität Heidelberg, Ludwigshafen, Germany; 4 AstraZeneca, Medical Department, Wedel, Germany; 5 Herz- und Diabeteszentrum Nordrhein-Westfalen in Bad Oeynhausen, Universitätsklinik der Ruhr Universität, Bochum, Germany

## Abstract

**Background:**

Patients with type 2 diabetes are at an increased risk for disease and treatment related complications after the initial approach of oral mono/dual antidiabetic therapy has failed. Data from clinical practice with respect to this patient group are however scarce. Therefore we set up a registry in primary care documenting the course and outcomes of this patient group.

**Methods:**

Diabetes Treatment Patterns and Goal Achievement in Primary Diabetes Care (DiaRegis) is a prospective, observational, German, multicenter registry including patients with type-2 diabetes in which oral mono/dual antidiabetic therapy has failed. Data were recorded at baseline and will be prospectively documented during visits at 6 ± 1, 12 ± 2 and 24 ± 2 months. The primary objective is to estimate the proportion of patients with at least 1 episode of severe hypoglycemia within one year.

**Results:**

313 primary care offices included 4,048 patients between June 2009 and March 2010 of which 3,810 patients fulfilled the in- and exclusion criteria. 46.7% of patients were female; patients had a median diabetes duration of 5.5 years and most were obese with respect to BMI or waist circumference. Hb^A1c ^at baseline was 7.4%, fasting plasma glucose 142 mg/dl and postprandial glucose 185 mg/dl. Co-morbidity in this patient population was substantial with 17.9% having coronary artery disease, 14.4% peripheral neuropathy, 9.9% heart failure and 6.0% peripheral arterial disease. 68.6% of patients received oral monotherapy, 31.4% dual oral combination therapy. The most frequent antidiabetic agent used as monotherapy was metformin (79.0%) followed by sulfonylureas (14.8%).

**Conclusions:**

DiaRegis is a large, prospective registry in primary diabetes care to document the course and outcomes of patients with type-2 diabetes in which the initial approach of oral mono/dual antidiabetic therapy has failed. The two year follow-up will allow for a prospective evaluation of these patients during multiple adjustments of therapy.

## Background

Patients with type 2 diabetes are at an increased risk for disease and treatment related complications. This is particularly true at a point where patients are switched from oral monotherapy, to combination drug therapies, diabetes is usually more advanced, complicated by a number of co-morbid disease conditions and the likelihood of disease and treatment related complications is increased. Intensive glucose control--in particular when complex insulin strategies are used--is associated with a 5-fold increased risk for severe hypoglycemia, which could induce harm in some patients [1].

The recent guideline of the German Diabetes Society (Deutsche Diabetes Gesellschaft, DDG) recommends to combine metformin with a number of different oral antidiabetic drugs or a GLP-1 analogue in patients whose Hb_A1c _remains ≥ 6.5% but is < 7.5% after 3-6 month of metformin monotherapy treatment (with nutritional counselling and sports). Insulin should be added if Hb_A1c _is still ≥ 6.5% after 3-6 month treatment with combination therapy or if Hb_A1c _is ≥ 7.5% after the initial metformin/other OAD monotherapy [2].

Of particular importance when optimizing pharmacotherapy is the balance between optimal blood glucose adjustments (Hb_A1c_, postprandial glucose) and the risk of hypoglycemia, the potential increase in body weight observed specifically with sulfonylureas, glinides and insulin regimens.

### Hb_A1c _adjustment

The primary target of guideline recommended therapies is the adjustment of Hb_A1c _as the ultimate therapeutic goal. Guidelines use Hb_A1c _as a surrogate in a lack of hard end points for most antidiabetic treatments [2]. This approach however is surprising since patients with type 2 diabetes whose Hb_A1c _was reduced from 8 to 7% in the UKPDS (United Kingdom Prospective Diabetes Study) did not exhibit a reduction in cardiovascular events [3]. That Hb_A1c _may only be an incomplete surrogate for cardiovascular endpoints was recently confirmed by the results of ACCORD (Action to Control Cardiovascular Risk in Diabetes, [4]), ADVANCE (Action in Diabetes and Vascular Disease: Preterax and Diamicron Modified Release Controlled Evaluation, [5]) and VADT (Veterans Affairs Diabetes Trial) [6]. It was found that intensive blood sugar lowering had no significant benefit in terms of decreasing cardiovascular risk. For new antidiabetic treatments the FDA issued guidelines requesting specific prospective analysis of the submitted data to assess cardiovascular safety and gives directions to the patient population to be entered in terms of cardiovascular comorbidity [7].

### Postprandial Glucose

This rise and fall of postprandial glucose level is mediated by the first-phase insulin response, in which large amounts of endogenous insulin are released, usually within 10 min, in response to nutrient intake. In individuals with type 2 diabetes, the first-phase insulin response is severely diminished or absent, resulting in persistently elevated postprandial glucose throughout most of the day [8]. Although there is still some debate whether postprandial glucose is a proper measure of overall glucose control, a number of studies have demonstrated a close correlation between postprandial glucose and glycaemic control [9-14]. Regardless of whether postprandial glucose is a better predictor of Hb_A1c _than fasting/preprandial glucose, most researchers agree that the best predictor of Hb_A1c _is mean blood glucose, which is a composite of both fasting/preprandial and postprandial glucose. It is reasonable to assume, that achieving near-normal postprandial glucose levels without risk for hypoglycemia is essential to achieving overall glycaemic control.

### Hypoglycemia

**Figure 1:**
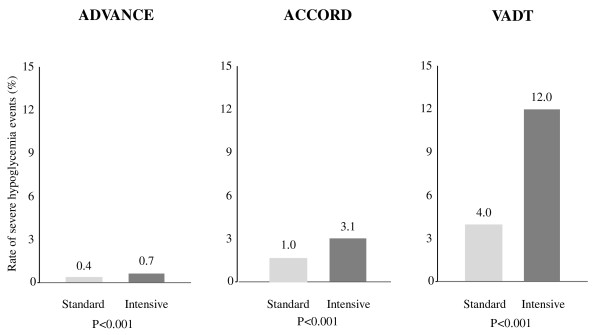
**Intensified treatment has been associated with an increase in the rate of hypoglycemia **[4-6].

Patients with blood glucose values less than 2.22 mmol/l are regarded, irrespective of symptoms, as being hypoglycaemic. Values between 2.22 and 2.78 mmol/l are still regarded as hypoglycaemic if cerebral symptoms of low blood glucose are evident. With many antidiabetic drugs such as sulfonylureas or insulin intensified blood glucose lowering has been associated with an increase in the rate of severe hypoglycemia (Figure 1) [4-6,15]. Severe hypoglycemia has been made responsible for excess deaths in the ACCORD trial [16]. Nineteen of the 41 excess deaths from cardiovascular causes in the study were attributed to "unexpected or presumed cardiovascular disease," which may plausibly be related to or may have been precipitated by hypoglycemia and misclassified as having a cardiovascular cause. Combination therapies, such as a sulfonylurea with insulin, are known to be associated with an increased risk for hypoglycemia and appear to have been used routinely in this study. Hypoglycemia not requiring medical assistance is not trivial however. In these cases a substantial reduction of cognitive and motor function as well as sympathetic counterregulation is observed [17].

### Weight gain with antidiabetic drugs

A positive correlation between weight gain and glycaemic control is well known [18]. Inappropriate weight gain has been demonstrated in landmark diabetes studies with insulin or oral anti-diabetic drugs [19]. Weight gain is associated with accelerated deterioration of beta-cell function in type 2 diabetes, and increases blood pressure and lipid levels in both type 1 and type 2 diabetes. Concerns about increasing weight may be a barrier to initiation or to intensification of insulin therapy. Insulin introduction may be delayed in type 2 diabetes, and patients may under-dose insulin to avoid weightgain [20].

### Costs

There are only three studies on the costs of diabetes in Germany [21-23]: In the CODE-2 Study (Costs of Diabetes in Europe-Type 2) the total expenses for type 2 diabetes were evaluated [22]. The costs per patient - compared to the average expenses for health insured patients - increased with complication state from the 1.3-fold (no complications) up to the 4.1-fold (macro- and microvascular complications). The second study was the CoDiM (Cost of Diabetes Mellitus) study [23,24]. Analysis of cost components revealed that the high costs associated with the care of diabetic patients could be largely attributed to inpatient care and overall medication costs. The third study was the German multi-centre, retrospective epidemiological cohort study ROSSO [21]. Total costs of diabetes care was 1,288 € for the first treatment year with diagnosed diabetes and rose to 3,845 € in year seven. Costs for treating complications dominated already in the first year after diagnosis. The mean direct treatment costs amounted to 3,210 € per patient and year for the first 6.5 years.

### Rationale

Treatment decisions in primary diabetes care are primarily based on evidence from randomized controlled trials. These trials however are confined by strict in- and exclusion criteria and patients seen in daily practice differ from the ones documented in clinical trials. In this respect the advantage of registries is to document a variety of patient types and physicians' treatment decisions without influence imposed by the study design. Therefore the external validity of findings in respect to real life conditions is usually high.

With respect to the disease and treatment related complications outlined, we think it is utmost important to gain an understanding of what the patient characteristics are, which disease related laboratory values patients have, what co-morbid diseases are present, what treatment options are used and which complications (e.g. cardiovascular events, hypoglycemia, weight gain) occur during the course of treatment. It is further important to gain an understanding of patient self reported outcomes (PRO) such as treatment satisfaction and health status.

## Methods

*Diabetes Treatment Patterns and Goal Achievement in Primary Diabetes Care *(DiaRegis) is a prospective, observational, national, multicenter registry with a follow-up of 2 years conducted in Germany. Data were recorded at baseline and will be prospectively documented during follow up visits at 6 ± 1, 12 ± 2 and 24 ± 2 months.

This registry is conducted in accordance with the ethical principles that have their origin in the Declaration of Helsinki and adhere to the principles of Good Epidemiology Practice (GEP), and applicable regulatory requirements. The protocol of this registry was approved by the ethics committee of the Landesärztekammer Thüringen in Jena, Germany on March 4^th ^2009. Patients that were enrolled into this registry provided written informed consent. It was further registered with the database of the *Verband forschender Arzneimittelhersteller *(VFA) [25].

### Research Question/Hypothesis

The aim of the present registry is to evaluate the specific characteristics, treatment patterns, quality of life and diabetes related events of type-2 diabetic patients who failed oral mono- or dual antidiabetic therapy and physicians deemed a change of therapy to be necessary. Because of the increasing risk of hypoglycemia after intensifying antidiabetic treatment when achieving near normal blood glucose values, we chose as a primary objective of DiaRegis to determine the proportion of patients with at least 1 episode of severe hypoglycemia within one year.

Secondary objectives were: 1) To evaluate the number of patients with at least 1 episode of severe, moderate or mild hypoglycemia after 1 and 2 years respectively; 2) To evaluate the number of hypoglycaemic events per patient after 1 and 2 years, respectively; 3) To evaluate the glycaemic profile; 4) To evaluate a change in measured body weight over the course of the study; 5) To evaluate fasting blood glucose and postprandial glucose of patients; 6) To evaluate the health status (EQ-5D) and treatment satisfaction (DTSQ); 7) To evaluate co-morbid disease conditions; 8) To determine costs associated with hypoglycemia; 9) To determine costs associated with therapy/treatment and disease related complications.

### Definitions

Hypoglycemia was classified as follows. In case of severe hypoglycemia the patient seeks medical attention or is admitted to hospital because of hypoglycemia. In case of moderate hypoglycemia patients experience symptoms of hypoglycemia and require assistance from a second person (a relative or friend, etc.), but no medical attention is necessary. Mild hypoglycemia is determined from blood glucose measurements (< 2.22 mmol/l; 40 mg/dl in any case; 2.22-2.78 or 50 mg/dl in case of symptoms) and is manageable without foreign help.

### Physician selection

Physicians have been selected based on a conditioned random sampling method. For this purpose a physician database with about 9.350 office based physicians (general practitioners, internists, practitioners and diabetologists) who treat patients with type 2 diabetes were asked in writing, to evaluate the possibility of participation. For this purpose physicians were requested to complete a questionnaire to check on whether they fulfil criteria for participation. Only physicians with at least 150 patients with type 2 diabetes under regular medical care were eligible to ensure, that a broad treatment spectrum will be covered and to secure that physicians were able to recruit patients fast and efficient and to receive a random distribution across all German regions. The sampling strategy should thus provide a representative dataset for the description of oral antidiabetic treatment patterns in Germany.

Physicians were contacted once and informed about the background and aims of the study. They were asked to complete a fax questionnaire indicating their eligibility and consent to participate in the study (or to give a reason for non-participation). The response further included site information about personal data as well as equipment of the site, especially with respect to the registry requirements. Non-participants will be assessed for comparability with participating physicians regarding physician and office characteristics.

1-2 weeks prior to the study, participating physicians received detailed information material, physician and patient questionnaires and access to a secure website for the entry of patient documentation. A telephone hotline was provided by the CRO and by the *Institut für Herzinfarktforschung*.

### Patient selection

Inclusion Criteria were as follows: 1) Patients with type-2 diabetes; 2) Age ≥ 40 years; 3) The treating physician indicated that blood glucose lowering therapy needed to be stepped up or changed, e.g. because glycaemic targets were not met or medication not safe and/or not tolerated; 4) Oral mono- or dual combination therapy (no insulin/no GLP-1 analogue); 5) The physician actually added another drug/switches therapy; 6) Provision of patient informed consent.

The following exclusion criteria applied: 1) Patients not under regular supervision of the treating physician for the duration of the study; 2) Patients with type 1 diabetes; 3) Pregnancy (gestational diabetes); 4) Diabetes secondary to malnutrition, infection or surgery; 5) Maturity onset diabetes of the young; 6) Known cancer or limited life expectancy; 7) Further reasons for exclusion were conditions that made it impossible or highly problematic for the patient to participate and to come to the follow-up visits (such as poor German language skills, serious disabilities or diseases, hospitalization) and acute emergencies; 8) Participation in a clinical trial.

The following subgroups are pre-defined and will be further explored: 1) patients that are prescribed insulin (with or without OAD) as the next step; 2) patients that are prescribed any combination with a DPP-4 inhibitor; 3) patients that are prescribed a combination of metformin and sulfonylureas; 4) patients that are prescribed a combination of metformin and a thiazolidinedione; 5) patients that are prescribed a combination of sulfonyureas and a thiazolidinedione; 6) patients that are prescribed any other combination including those receiving a GLP-1 analogue/mimetic.

### Documented variables

**Figure 2:**
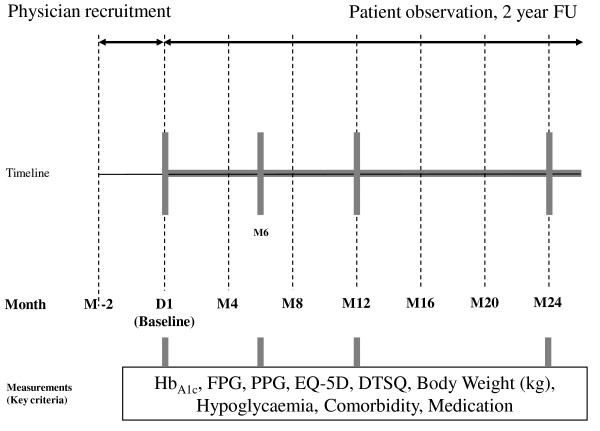
**Graphical study design**. M, month(s); FU, follow-up; D, Day; FPG, fasting plasma glucose; PPG, post-prandial glucose; EQ-5D, EuroQol-5D; DTSQ, Diabetes Treatment Satisfaction Questionnaire

Table 1 gives an overview of variables obtained during regular visits at baseline and during follow-up including those from the physicians and patient questionnaires. Figure 2 illustrates the study design. Further to these variables patients received a patient diary for the documentation of hypoglycaemic events (date, measured blood glucose, severity of hypoglycemia, glucagon use) during the 2 year follow-up.

**Table 1:** Study flow chart

Evaluation	Observational Phase			
Month	M0	M6 (± 1 M)	M12 (± 2 M)	M24 (± 2 M)
Demographics & Co-morbidity				
Inclusion/Exclusion Criteria	X			
Patient Identification	X	X	X	X
Date of visit	X	X	X	X
Date of consent	X			
Demographics	X			
Physical examination	X	X	X	X
Diabetes history	X			
Diabetic complications and co-morbidity	X	X	X	X
Pharmacotherapy	X	X	X	X
Laboratory values:				
TC, TG, HDL-C, LDL-C	X	X	X	X
Serum-Creatinine, Micro-, Macroalbuminuria	X	X	X	X
CRP	X	X	X	X
Hb_A1c_, FPG, PPG	X	X	X	X
Non-drug intervention	X	X	X	X
Hypoglycaemic events	X	X	X	X
Body Weight				
Body weight and height	X	X	X	X
Hip and waist circumference	X	X	X	X
EQ-5D, DTSQ	X		X	X
Hypoglycemia Awareness Questionnaire	X	X	X	X
WHO-5	X		X	X

TC, total cholesterol; TG, triglycerides; HDL, high density lipoprotein; LDL, low density lipoprotein; Hb_A1c_, glycosylated haemoglobin A1c; CRP, C-reactive protein; FPG, fasting plasma glucose; PPG, post-prandial glucose; EQ-5D, EuroQol-5D; DTSQ, Diabetes Treatment Satisfaction Questionnaire; WHO, World Health Organization

### Monitoring

To reduce the logistics of coordinating such a nation-wide study, participating physicians received no other instructions than the information material sent to them by mail prior to the survey. Further they were contacted within a few days prior to commencement of the study to ensure compliance with the registry protocol. To control for a proper procedure and to ensure the robustness of the data obtained, a specifically adapted monitoring system was designed to meet the logistic needs. This monitoring concept included telephone and fax monitoring and random on-site visits. The monitors were specifically trained prior to start of the study, especially in terms of survey procedures and criteria for follow-up appointments.

On-site visits were performed within the month following the recruitment into the registry, with a focus on quality of data and measurements. Monitoring visits are conducted in at least 10% of the participating sites randomly selected. Monitoring is split into 4 parts suggesting to monitor 3% of the sites (n = 13) after enrolment of patients, 3% after 6 months (n = 13), 2% after 12 months (n = 9) and 2% after 24 months (n = 9). The observed quality problems and errors at the monitored sites allow an estimation of the overall error rate of the study. For every site monitored selected patient data were to be checked for integrity and compared with the source data. The physician or physician assistants were requested to rectify observed errors.

### Database Methodology

Data entry was performed by the physician or study nurse via a secure website directly into an electronic database. This approach allowed online checks for plausibility and integrity.

Data from the patient questionnaire (paper version) which was asked to be completed by the patient during the visit were transferred to the CRO appointed. The questionnaires were scanned and transferred to the *Institut für Herzinfarktforschung *for evaluation.

All data sets were checked for incorrect data and corrected if applicable; all corrections are documented. All data sets are submitted for biostatistic analysis.

### Statistical Methods

Descriptive statistics (mean, standard deviation, range, percentiles, proportions etc.) are calculated for all patients and the pre-specified subgroups. Chi square tests or Wilcoxon rank sum tests are used where appropriate to investigate differences in distributions of variables by subgroups. Multivariable analyses are conducted as necessary to adjust for other covariates. All statistical analyses are conducted using the statistical software package SAS 9.1 (SAS Institute, Cary, NC, USA).

The analysis is planned to: 1) Describe the baseline patient characteristics, including risk factors, co-morbidity, pharmacotherapy received, switching between therapeutic options and a number of PRO measures; 2) Estimate the incidence and frequency of hypoglycaemic events after 1 and after 2 years, as well as changes in PRO measures at each visit; 3) Analyse any associations between the choice of pharmacotherapy and outcomes after 1 and after 2 years; 4) Identify independent determinants of hypoglycaemic events using multivariable statistics.

A specific objective of DiaRegis is to estimate the cost of hypoglycemia. Therefore it will consider the number of patients with events and the number of events per patient.

### Sample Size

The primary objective for sample size consideration is to estimate the proportion of patients with at least 1 episode of severe hypoglycemia within one year. Based on recently reported rates of severe hypoglycaemic events between 0.4% and 3.1% per year, a conservative estimation of the overall rate of severe hypoglycaemic events of 1.5% over 1 year and a desirable precision of the estimate of ± 0.337% the sample size had to be 5000 patients. In case of a reduced recruitment of 3,900 patients or 3,600 patients an estimate precision of ± 0.382% and ± 0.397% was expected.

## Results

**Figure 3:**
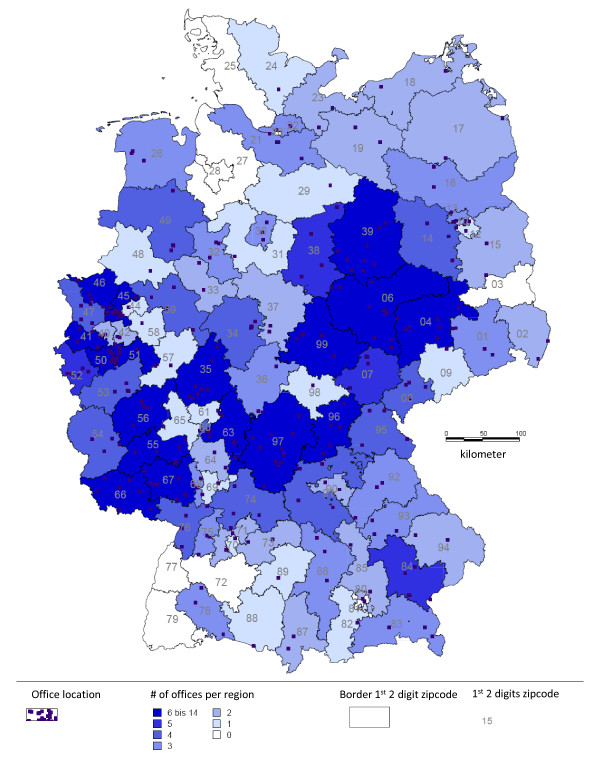
**Regional distribution of participating practices throughout Germany (as of 30.04.2010)**.

**Figure 4:**
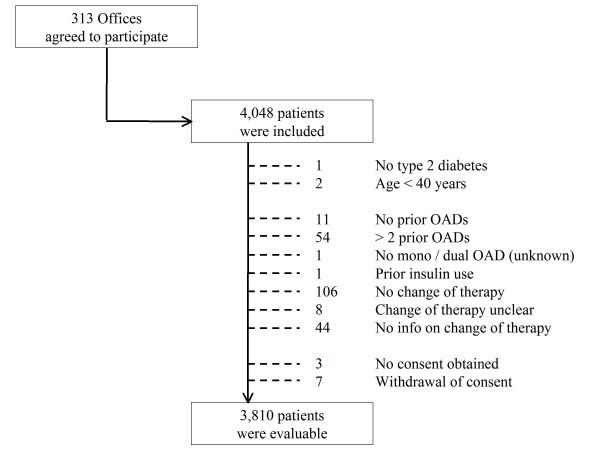
**Patient flow chart**. OAD, oral antidiabetic drugs

313 primary care offices included patients into DiaRegis. 109 were general practitioners, 116 diabetologists, 84 internists and 4 others (Figure 3). Between June 2009 and March 2010 these physicians included a total of 4,048 patients. Of these 238 patients were excluded because in- and exclusion criteria were not met, including 7 patients who withdrew informed consent. Therefore 3810 patients out of 313 practices were available for the description of baseline patient characteristics (Figure 4).

### Patient characteristics and laboratory values at baseline

Of the 3810 patients available for the baseline analysis 46.7% were female; patients had median diabetes duration of 5.5 years and most were obese with respect to BMI or waist circumference. Hb_A1c _at baseline was 7.4%, fasting plasma glucose 142 mg/dl and postprandial glucose 185 mg/dl (for further baseline characteristics see Table 2). In comparison men were slightly younger, and had higher blood glucose values (Hb_A1c_, fasting and postprandial glucose) along with significant differences in lipid values (p < 0.0001).

**Table 2:** Patient characteristics and laboratory values at baseline

	Total (n = 3,810)	Men (n = 2,033)	Women (n = 1,777)	p-value (m vs. f)
Age (years)	65.9 (57.6-72.9)	64.7 (56.8-72.0)	67.2 (58.6-74.1)	< 0.0001
Diabetes duration (years)	5.5 (2.9-9.4)	5.5 (2.8-9.3)	5.6 (3.0-9.4)	n.s.
BMI (kg/m^2^)	30.0 (27.0-35.0)	30.0 (27.0-34.0)	31.0 (28.0-36.0)	< 0.0001
Waist circumference (cm)	106 (98-116)	108 (100-118)	104 (95-114)	< 0.0001
Total cholesterol (mg/dl)	205 (177-232)	199 (172-228)	211 (185-236)	< 0.0001
HDL-Cholesterol (mg/dl)	47 (40-57)	44 (38-52)	52 (44-61)	< 0.0001
LDL-Cholesterol (mg/dl)	120 (98-145)	116 (93-142)	123 (103-147)	< 0.0001
Triglycerides (mg/dl)	176 (127-243)	184 (129-266)	169 (125-228)	< 0.0001
Hb_A1c _(mg/dl)	7.4 (6.8-8.3)	7.5 (6.9-8.4)	7.3 (6.8-8.1)	< 0.0001
Fasting plasma glucose (mg/dl)	142 (119-171)	144 (121-175)	140 (118-168)	< 0.01
Postprandial glucose (mg/dl)	185 (155-221)	189 (159-225)	179 (151-216)	< 0.0001

BMI, body mass index; HDL, high density lipoprotein; LDL, low density lipoprotein; Hb_A1c_, glycosylated haemoglobin A1c

### Risk factors and co-morbid conditions at baseline

11.9% of patients were smoking and 62.9% were drinking any alcohol (men > women; p < 0.0001; Table 3). On the other hand more men were doing any sports even if seldom (45.0 vs. 34.6%; OR 1.55; 95%CI 1.35-1.78). While the prevalence of coronary artery disease (23.4 vs. 11.7%; OR 2.31; 95%CI 1.93-2.76) and myocardial infarction (38.9 vs. 24.1% of those with CAD; OR 2.00; 95%CI 1.38-2.92) was higher in men than in women, clinical evidence of relevant depression (indicated by the treating physician) was less frequent in men (3.6 vs. 7.4%; OR 0.47; 95%CI 0.35-0.63; p < 0.0001).

**Table 3:** Risk factors and co-morbid conditions at baseline

	Total (n = 3,810)	Men (n = 2,033)	Women (n = 1,777)	OR (95%CI)	p-value
Smoker	11.9	14.4	9.1	1.69 (1.37-2.08)	< 0.0001
Any alcohol, even rare	62.9	79.9	43.6	5.15 (4.38-6.06)	< 0.0001
Any Sports, even rare	40.1	45.0	34.6	1.55 (1.35-1.78)	< 0.0001
Dyslipidemia	63.3	64.7	61.6	1.14 (1.00-1.31)	< 0.05
Hypertension	84.4	83.6	85.3	0.88 (0.74-1.05)	n.s.
Malignancy	2.0	1.8	2.2	0.83 (0.52-1.30)	n.s.
Coronary artery disease	17.9	23.4	11.7	2.31 (1.93-2.76)	< 0.0001
Prior MI	34.4	38.9	24.1	2.00 (1.38-2.92)	< 0.001
Prior stroke/TIA	4.6	5.0	4.2	1.18 (0.87-1.60)	n.s.
PAD	6.0	7.7	4.1	1.95 (1.46-2.60)	< 0.0001
Amputation*	0.9	1.2	0.6	2.20 (1.05-4.60)	< 0.05
Heart failure	9.9	10.4	9.3	1.13 (0.91-1.40)	n.s.
Autonomous neuropathy	3.4	3.7	2.9	1.28 (0.89-1.84)	n.s.
Symptoms of peripheral neuropathy	14.4	15.7	12.8	1.27 (1.06-1.53)	< 0.05
NPDR	3.8	3.6	3.9	0.91 (0.65-1.28)	n.s.
PDR	0.5	0.5	0.5	0.97 (0.39-2.40)	n.s.
Blindness	0.2	0.2	0.2	1.17 (0.26-5.22)	n.s.
Clinically relevant depression	5.4	3.6	7.4	0.47 (0.35-0.63)	< 0.0001

PDR, proliferative diabetic retinopathy; MI, Myocardial Infarction; NPDR, non-proliferative diabetic retinopathy; PAD, peripheral arterial disease; TIA, transitory ischemic attack; OR, odds ratio; CI, confidence interval; * toe, foot or lower extremity

### Drug utilization

**Figure 5:**
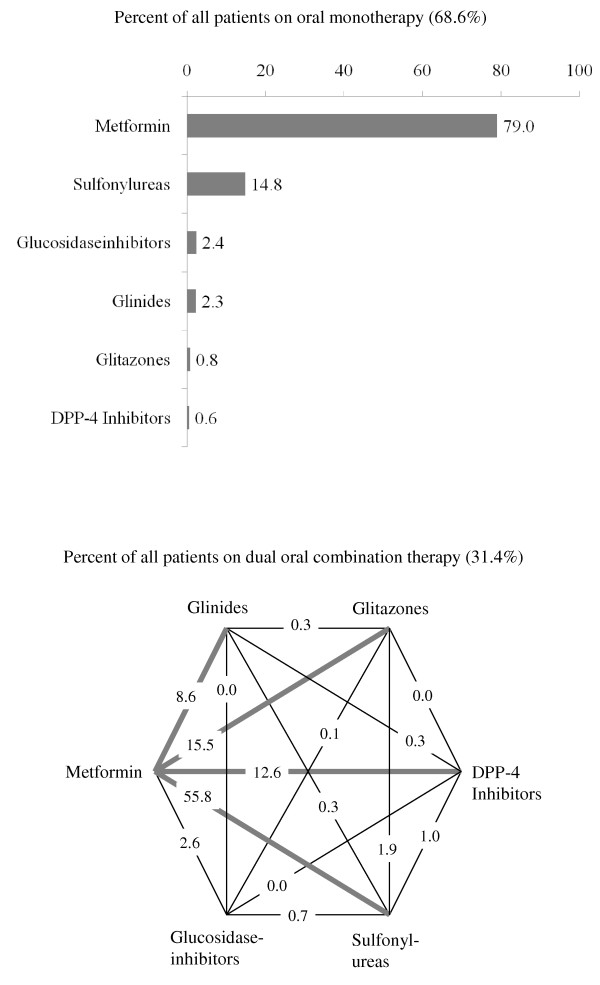
**Drug utilization in DiaRegis of patients on oral monotherapy (68.6% of all patients) (panel A) and on dual oral combination therapy (31.4%of all patients) (panel B)**.

68.6% of patients received oral monotherapy, 31.4% dual oral combination therapy. The most frequent antidiabetic agent used in monotherapy was metformin (79.0%) followed by sulfonylureas (14.8%) (Figure 5, **panel A**). In patients with dual oral combination therapy the most frequent combinations were metformin with sulfonylureas (55.8%), glitazones (15.5%), DPP-4 inhibitors (12.6%) and glinides (8.6%), respectively (Figure 5, **panel B**). Differences between male and female patients were negligible and not significant except for a slightly more frequent use of glinides in men (10.1 vs. 6.8%; p < 0.05).

**Figure 6:**
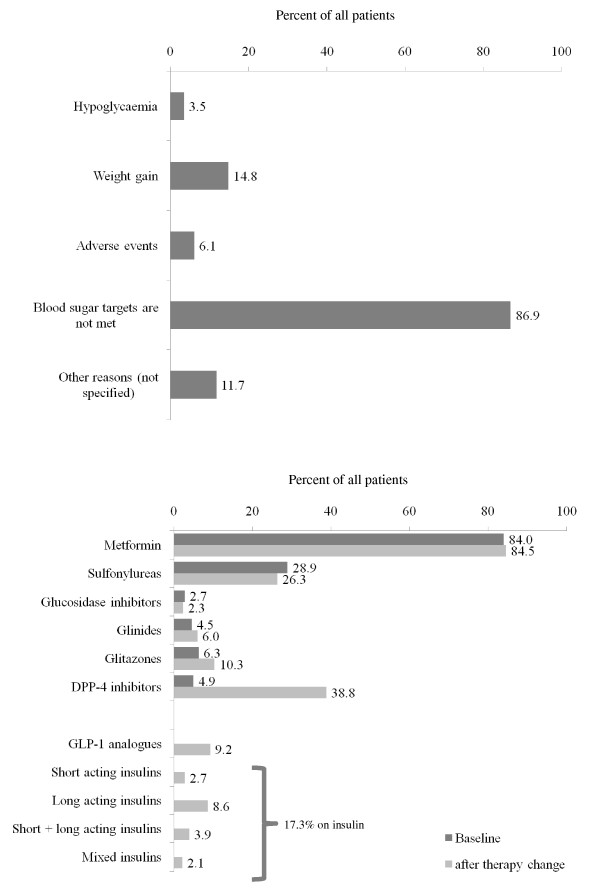
**Reason for a change of pharmacotherapy at baseline (panel A) and choice of drugs thereafter (panel B)**.

At the inclusion visit patients' oral antidiabetic agents were either adjusted, added, substituted for another oral antidiabetic agent or insulin or a GLP-1 analogue was added. Being asked which reason led to this decision the physicians indicated that not meeting blood glucose targets was the most frequent reason (86.9%). Hypoglycemia, weight gain, adverse events and unclassified reasons were much less important (Figure 6, **panel A**). After therapy change the use of sulfonylureas was slightly reduced (26.3 vs. 28.9%), while the use of DPP-4 inhibitors increased significantly (38.8 vs. 4.9%). Newly introduced were GLP-1 analogous (9.2%) and insulin (17.3%) (Figure 6, **panel B**). Antidiabetic drug therapy was not statistically different between men and women at baseline. After therapy change only the use of metformin (85.6 vs. 83.3%; OR 1.19; 95%CI 1.00-1.42) and glitazones (11.6 vs. 8.8%; OR 1.35; 95%CI 1.09-1.67) was higher in men.

## Discussion

DiaRegis is a large prospective registry evaluating disease and treatment related complications in patients with type-2 diabetes in Germany. These data supplement evidence obtained from randomized controlled trials by documenting drug use in specific patient populations, concomitant diseases, concurrent pharmacotherapies and are thus highly relevant for daily practice.

### Need for a prospective observational registry

There are a number of epidemiological studies which documented (among other aspects) the treatment of type-2 diabetes. Table 4 gives an overview about studies closely related to the research question of DiaRegis. These included the 2001 HYDRA study [26], the 2003 DETECT study [27] and the studies CODE-2 [22,28,29], ROSSO [21,30], DUTY [31], CoDiM [23] and DIG [32,33]. These have aimed at describing the situation of diabetic patients in primary care in Germany. Finally SWEETHEART is a prospective registry in patients with type-2 diabetes and acute myocardial infarction with or without ST elevation within the previous 24 h. It started in October 2006 and included 2,772 patients in 31 hospitals in Germany but results have been presented [34] but not published yet.

**Table 4:** Comparison of DiaRegis with other existing registries (part I)

	DiaRegis	CODE-2	CoDiM	ROSSO
Reference		[22]	[23,24]	[21]
No. of physicians	313	135	n.a.	192
No. of patients	3,810	809	26,971	3,286
Recruitment	06/2009-03/2010	1 year (1998)	2001	1995-2003
Follow-up	2 years	none	none	Min. 4 years
Design	Prospective cohort	Retrospective, cross-sectional	Database analysis	Retrospective cohort
Monitoring for data verification	Yes (10%)	None	None	None
Proportion T2D	100%	100%	100%	100%
Patients	Patients on oral mono- or dual antidiabetic combination therapy	Patients with type-2 diabetes mellitus	Patients with type-2 diabetes mellitus	Patients with newly diagnosed type-2 diabetes mellitus
Median age (years)	65.9	66	n.a.	Mean 62.5 ± 9.6
Female (%)	46.7	52	n.a.	51.4
BMI (median)	30.0	28	n.a.	Mean 29.8 ± 5.1
Focus	Hypoglycemia incidence with antidiabetic drug use	Costs of type-2 diabetes	Costs of diabetes	Costs of diabetes

n.a., not available

Most of these studies however either had a detailed look on the prevalence of type-2 diabetes in primary care practice (HYDRA and DETECT), the co-morbidity burden (DETECT), the costs (CODE-2, CoDiM and ROSSO), on self-monitoring of blood glucose (ROSSO) or on the effect of tailored intervention on target achievement (DUTY, DIG). Most of these did not consider nutritional/dietary aspects, as well as a more customized approach to the recruited patients' lifestyle and physical activity [35,36]. Some were also retrospective in design (CODE-2, CoDiM and ROSSO). Moreover most epidemiological studies are rather old, and contemporary data are scarce [32,33].

The particular value of DiaRegis relates to the prospective evaluation of antidiabetic therapy in patients being prone to disease and treatment related complications such as hypoglycaemia. The prospective design will improve data quality in comparison to other registries because a structured assessment and documentation is usually more complete and consistent than retrospective analyses of existing data which have been obtained for other purposes. With this respect the monitoring concept in this registry is important (tables 4 and 5), in that the validation of data with the respective source data will further improve data quality. This has previously been shown to be effective in other registries [37,38].

**Table 5:** Comparison of DiaRegis with other existing registries (part II)

	HYDRA	DETECT	DUTY	DIG
Reference	[26,39]	[27]	[31]	[32,33]
No. of physicians	1,912	3,188	n.a.	238
No. of patients	45,125	55,518	59,035	4,020
Recruitment	09/2001	09/2003	2001-2003	2002-2004
Follow-up	none	1 and 4 years	9 months	4 years
Design	Cross-sectional, no follow-up	Cross-sectional with a prospective follow-up of a subgroup	Prospective cohort study	Prospective cohort study
Monitoring for data verification	None	None	None	None
Proportion T2D	16.1%	14.1%	100%	100%
Patients	Any patient in primary care	Any patient in primary care	Type-1 or type-2 diabetes mellitus	Type-2 Diabetes mellitus
Median age (years)	52.4	Mean 53.9 ± 17.3	64.4 ± 11.7	Mean 61.8 ± 8.1
Female (%)	60.0	59.2	50.9	46.8
BMI (median)	n.a.	Mean 26.8 ± 5.3	Mean 28.7 ± 4.8	Mean 30.7 ± 5.2
Focus	Prevalence of hypertension, diabetes and microalbuminuria	Co-morbidity burden of patients with diabetes	Effect of tailored intervention on target achievement	Application of guidelines in clinical practice

n.a., not available

## Conclusions

DiaRegis is a large, prospective registry in primary diabetes care to document the course and outcomes of patients with type-2 diabetes who failed the initial approach of oral mono/dual antidiabetic therapy. The two year follow-up will allow for a prospective evaluation of these patients during multiple adjustments of therapy. The results of this registry will help to understand how the degree of diabetes control and treatment-related events such as hypoglycemia or diabetes-related complications impact patient reported outcomes. It further might give insight in how to improve patient care in type 2 diabetes with the ultimate goal to improve outcomes and possibly prolong life.

## Competing interests

Peter Bramlage, Anselm K. Gitt and Diethelm Tschöpe have received research support and honoraria for lectures from a number of pharmaceutical companies including Bristol-Myers Squibb and AstraZeneca, the sponsors of this registry. Christiane Binz, Michael Krekler and Tanja Plate are employees of the sponsors. Evelin Deeg has no potential conflict of interest to disclose.

## Authors' contributions

PB, AKG, DT, CB, MK and TP have been involved in the conception and design of the study. ED is responsible for the analysis of data. PB has drafted the manuscript based on the protocol and all other authors have been revising the article for important intellectual content. All authors have finally approved the version to be published.
